# Mesonephric-like adenocarcinoma of the female genital tract: possible role of *KRAS*-targeted treatment—detailed molecular analysis of a case series and review of the literature for targetable somatic *KRAS*-mutations

**DOI:** 10.1007/s00432-023-05306-9

**Published:** 2023-09-05

**Authors:** Christine E. Brambs, Lars-Christian Horn, Ruth Hiller, Irene Krücken, Christian Braun, Corina Christmann, Astrid Monecke, Anne Kathrin Höhn

**Affiliations:** 1 Department of Obstetrics and Gynecology, Lucerne Cantonal Hospital, Spitalstrasse, 6000 Lucerne, Switzerland; 2 Division of Gynecologic, Breast and Perinatal Pathology, Institute of Pathology, University Hospital Leipzig, Leipzig, Germany; 3 Division Molecular Pathology, Institute of Pathology, University Hospital Leipzig, Leipzig, Germany

**Keywords:** Endometrium, Ovary, Mesonephric, Adenocarcinoma, *KRAS*-mutation, G12C-Targeted treatment, Sotarasib, Prognosis

## Abstract

**Purpose:**

Mesonephric-like adenocarcinomas (MLA) of the female genital tract represent a rare and relatively recently described neoplasm exhibiting characteristic morphologic and immunohistochemical findings commonly associated with a *KRAS*-mutation. Most cases display an aggressive clinical behavior, but knowledge about treatment approaches is limited, especially for targeting *KRAS*.

**Methods:**

We report a series of eight cases with a detailed molecular analysis for *KRAS*. These cases as well as the data of previously published cases with detailed information regarding *KRAS*-mutational events were reviewed for a potential targeted approach and its prognostic impact.

**Results:**

Both the uterine and ovarian MLA harbor a somatic *KRAS-mutation* in about 85% of the reported cases, affecting the hotspot codons 12 and 13. 15.7% of the endometrial and 15.6% of ovarian MLA are wild type for *KRAS*. A p.G12A-alteration was seen in 5.6% (5/89) of the endometrial and in 6.2% (2/32) of the ovarian tumors, for p.G12C in 7.9% and 6.2%, for p.G12D in 32.6% and 34.5% and for p.G12V in 36% and 37.5%, respectively. Very limited data are available regarding the prognostic impact of different mutational sites within the *KRAS-gene* without significant prognostic impact.

**Conclusion:**

Because of a specific p.G12C-*KRAS* somatic mutation, only the minority of MLA (7.9% with uterine and 6.2% with ovarian primary) are potentially targetable by sotarasib in that rare but aggressive subtype of adenocarcinoma of the female genital tract. Until now, the different location of a somatic *KRAS*-mutation is of no prognostic impact.

**Supplementary Information:**

The online version contains supplementary material available at 10.1007/s00432-023-05306-9.

## Introduction

Mesonephric-like adenocarcinoma (MLA) is a recently recognized malignancy of the female genital tract (Mirkovic et al. 2018; McFarland et al. 2016; McCluggage 2022). This morphologically distinct type of carcinoma arises both in the endometrium and the ovary with a characteristic immunophenotype [TTF-1^positive^/estrogen receptor^low/negative^ (Kim et al. 2021; Euscher et al. 2020; Horn et al. 2020; Mills et al. 2022)]. In 2021, Deolet et al. (2021) reviewed published cases and identified 115 endometrial and 39 ovarian tumors. MLA are very rare and account for about 1% of all endometrial cancers (Pors et al. 2018; Kolin et al. 2019; Ma et al. 2022; Kim et al. 2022). Since the original description, several studies reported an aggressive clinical behavior (Euscher et al. 2020) with unusual distant spread (Deolet et al. 2021; Al Nabhani et al. 2022), predominantly to the lungs (Pors et al. 2018; Euscher et al. 2020; Pors et al. 2021). Molecular analysis of the first reported cases identified a *KRAS*-mutation in MLA (Mirkovic et al. 2018), which was later confirmed by others (Horn et al. 2020; Euscher et al. 2020; Kim et al. 2021; Mills et al. 2022; Ma et al. 2022; Kim et al. 2022). Overall, about 80% of endometrial MLA harbor *KRAS*-mutational alterations (Horn et al. 2020; Euscher et al. 2020; Kim et al. 2021; Mills et al. 2022; Ma et al. 2022; Kim et al. 2022; Mirkovic et al. 2018) and up to one-third represent additional mutational events (Mirkovic et al. 2018; Na and Kim 2019; Kolin et al. 2019; Euscher et al. 2020; da Silva et al. 2021; Mills et al. 2022; Ma et al. 2022). The purpose of this study was to add cases of MLA with detailed molecular analyses to the existing literature and provide further data obtained from the literature for *KRAS*-molecular alterations which are potential therapeutic targets. Additionally, the prognostic impact of different sites of a *KRAS*-mutation was examined.

## Materials and methods

### Analyses of the cases from Leipzig University Hospital, Germany

All cases of the “Leipzig cohort” have been diagnosed since 2018, and the majority (5/8) resulted from second opinions and were sent in from outside institutions.

All cases were thoroughly analyzed for morphologic (McFarland et al. 2016; Euscher et al. 2020; Kim et al. 2022; McCluggage 2022) and immunohistochemical parameters (McFarland et al. 2016; da Silva et al. 2021; Ma et al. 2022; Mills et al. 2022; McCluggage 2022) consistent with mesonephric-like features. Additionally, NGS analyses for molecular alterations were performed in all cases (see Supplementary material).

### Evaluation of the previously published cases

Previously published cases of uterine and ovarian MLA were re-evaluated for molecular results focusing on *KRAS*-mutations (Mirkovic et al. 2018; Chapel et al. 2018; Patel et al. 2018; Na and Kim 2019; Yano et al. 2019; Kolin et al. 2019; McCluggage et al. 2020; Dundr et al. 2020; Horn et al. 2020; Euscher et al. 2020; Seay et al. 2020; da Silva et al. 2021; Deolet et al. 2021; Ma et al. 2022; Kim et al. 2021, 2022; Mills et al. 2022; Park et al. 2022; Al Nabhani et al. 2022; Deolet et al. 2022; Koh et al. 2022). Those cases were analyzed for the frequency of *KRAS*-mutation (wild type versus mutated). Within the cases with details of *KRAS*-mutational analyses, the specific site of the mutational event was recorded, overall and separated by the specific tumor site (uterine versus ovarian).

Furthermore, survival data were analyzed in correlation with the different localization of the *KRAS*-mutation to evaluate the prognostic impact of specific mutational sites.

This review includes all cases of mesonephric-like adenocarcinomaa of the female genital tract that have been published in English until early May 2023.

The duration from diagnosis to first event was calculated as the time between surgery/treatment until the first event, defined as recurrence, metastasis, or death of disease.

## Results

### Cases from Leipzig University Hospital

All cases from the "Leipzig cohort" displayed mixed morphologic features on H&E-staining. Almost all (87.5%; 7/8) had supportive immunohistochemical confirmation (positivity for at least either TTF-1, GATA-3, CD 10 (luminal staining) or calretinin, and negativity for estrogen receptor). One single case was negative for those immunomarkers but had a *KRAS*-mutational confirmation.

**Fig. 1:**
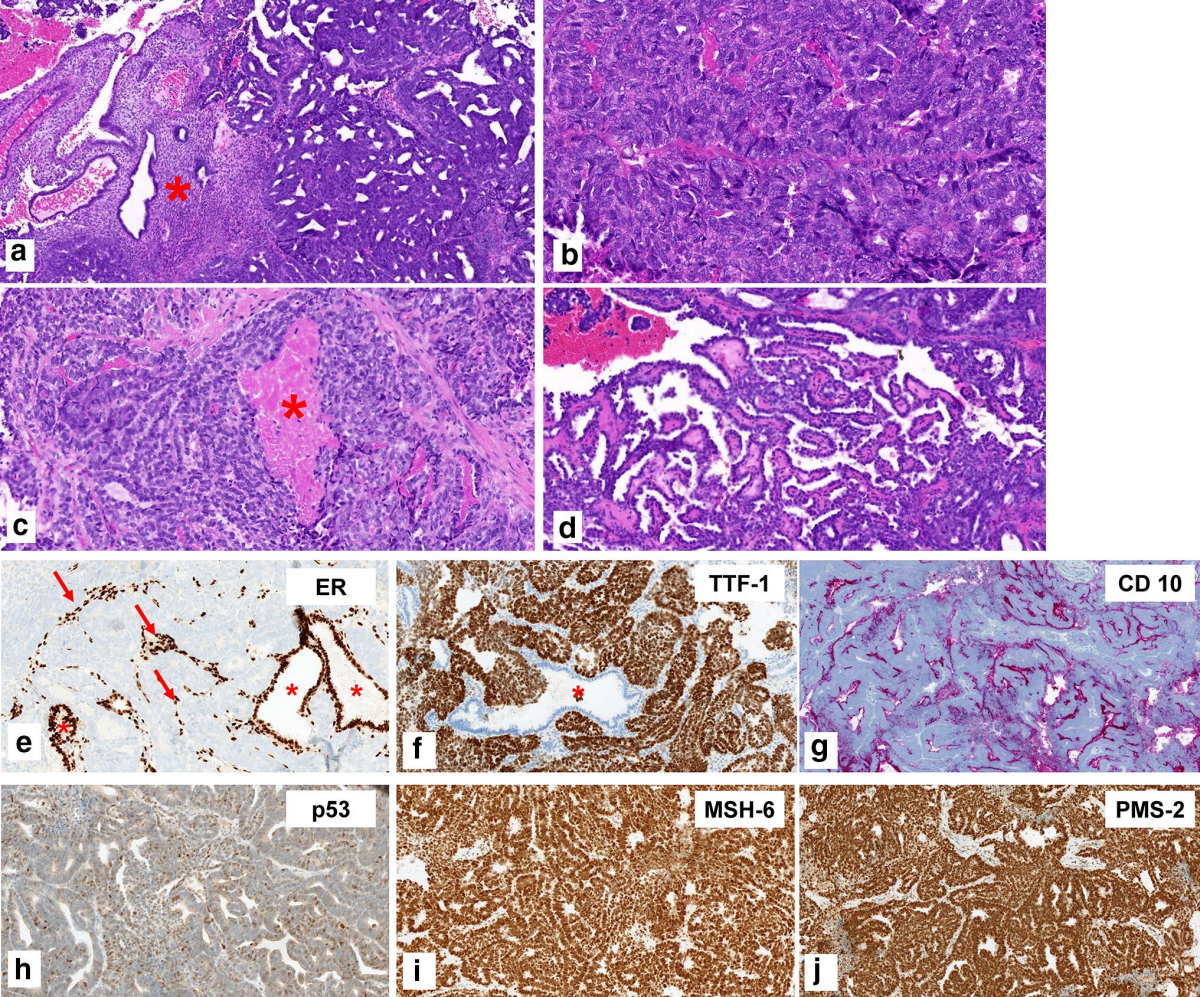
Histomorphologic and immunohistochemical features of mesonephric-like adenocarcinoma of the endometrium. **a** Atrophic non-neoplastic endometrium (*) and endometrioid-like pattern of the tumor (right side). **b** Solid pattern with some slit-like spaces. **c** Solid tumor growth with comedoid tumor necroses (*). **d** Papillary tumor growth. **e** Immunohistochemical staining against estrogen receptor: negative staining of the tumor cells but positive nuclear staining for endometrial stromal cells (arrows) and benign endometrial glands (*). **f** Strong and diffuse nuclear staining for TTF-1 within the tumor cells; benign endometrial glands are negative (*). **g** Apical positivity of the tumor cells for CD 10. **h** Nuclear wild-type staining of the tumor cells for p53. Retained nuclear staining for the mismatch-repair proteins MSH-6 (**i**) and PMS-2 (**j**)

Characteristic histopathologic and immunohistochemical findings are illustrated in Fig. 1. All cases showed a pathogenic *KRAS*-mutation. Three out of the eight cases displayed additional mutational alterations affecting CTNNB1, TP53, HFE, Jak-2, EGFR, and Her-2. All of them represented variants with unknown significance; see Table 1 for clinicopathologic characteristics.

**Table 1:** Mutational findings of mesonephric-like endometrial adenocarcinoma (MLA) of the endometrium (“Leipzig cohort”, please see text)

Case #	Age	Tumor size (cm)	Histologic subtype	*KRAS* mutational details			
				Amino acid change	Sequence change	Variant allele frequency (%)	Additional findings
1	74	5.5	Pure MLA	p.G12V	c.35G > T	58	–
2	54	4.0	Pure MLA	p.G12D	c.35G > A	77.6	–
3	64	3.2	Pure MLA	p.G12V	c.35G > T	52	–
4	61	4.7	Pure MLA	p.G12V	c.35G > T	34	–
5	57	1.1	Pure MLA	p.G12C	c.34G > T	56.6	–
6	52	2.8	Mixed MLA and endometrioid AC, G1	p.G12D	c.35G > A	50.2	Benign variants: *EGFR*, *Her-2*, *TP53*
7	64	5.1	Pure MLA	p.G12V	c.35G > T	94	Possibly pathogenic: *CTNNB1*, *HFE*, VUS: *Jak2*, benign variants: *ERBB2*, *TP53*
8	56	4.5	Pure MLA	p.G12D	c.35G > A	54.5	–

Follow-up data were available from one case (case 5). That patient presented with a hepatic recurrence after 11 months.

### Previously published cases

Some studies did not examine the *KRAS*-mutational status of their reported cases and focused on morphologic findings and/ or clinical features (Pors et al. 2021). Other studies performed molecular analyses but did not report the results in detail (Park et al. 2022).

Detailed molecular results were reported for 89 of the endometrial and 32 of the ovarian ML-AC (see Table S1).

Thirty cases of endometrial and 11 of ovarian MLA were informative regarding prognostic data in correlation to specific site of the *KRAS*-mutation (Supplementary Table S2).

Overall, 70% (21/30) of the patients with endometrial MLA recurred or died of the disease after a median time of 28.6 months (range 1–149 months). Within the patient group with an ovarian location of the MLA, 45.5% (5/11) recurred or died of the disease within 8.4 months (range 1–18 months).

**Fig. 2:**
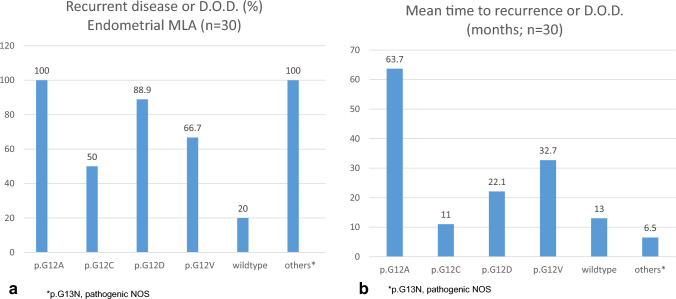
Frequency and mean time to recurrence or death of the disease (D.O.D.) of patients with endometrial MLA in correlation to different sites of mutational event within the *KRAS*-gene. For details, please see text and for detailed information Supplementary Table S1

For endometrial MLA, recurrence or death of the disease was reported for 66.7% (6/7) with a mean time of 32.7 months (range 9–100 months) of patients with a p.G12V-alteration, 88.9% (8/9) within a mean time of 22.1 months (range 1–84 months) with a p.G12D-alteration, 50% (1/2) within 11 months with p.G12C-alteration, 100% (3/3) within a mean of 63.7 months (range 18–149 months) with a p.G12A-alteration, and for 20% (1/5) within 13 months for those with *KRAS* wild type (Fig. 2). The two cases with a p.G13N-alteration and a pathogenetic *KRAS*-mutation with unknown localisation (NOS) recurred after 6.5 months (range 4–9 months).

Because of the limited data of the reported cases regarding follow-up, statistical analyses were not performed. The details are summarized in Supplementary Table S2.

### Mutational events of all cases ("Leipzig cohort" AND published cases)

Overall and including the eight endometrial MLA from the "Leipzig cohort", about one-third of the MLA harbored p.G12V- and p.G12D-*KRAS-mutations*. About 16% of the tumors were wild type. Only two endometrial MLA (2.2%; 2/89) were diagnosed with mutational events at p.G13N and p.G13G, resepectively.

**Fig. 3:**
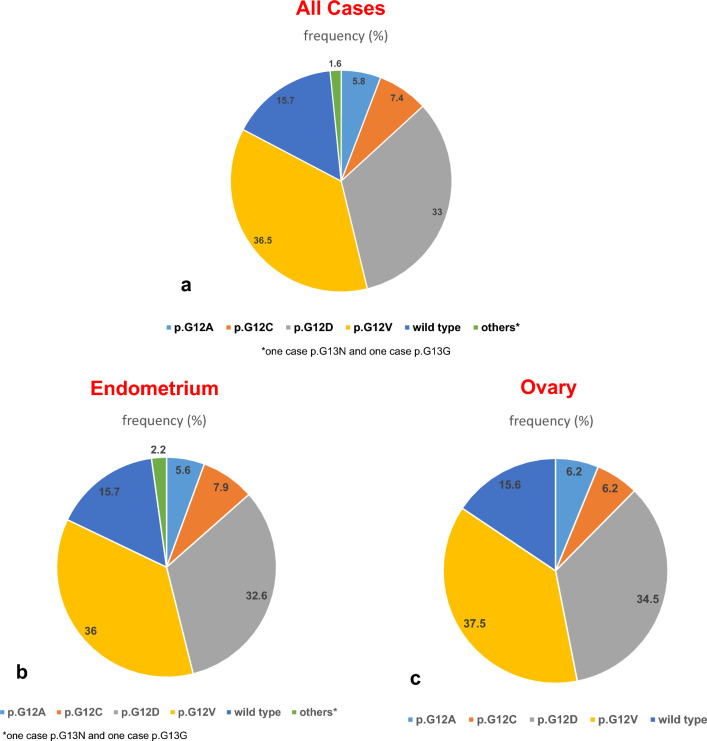
Mutational sites and the frequency of *KRAS*-alterations in mesonephric-like adenocarcinomas (ML-AC) of the female genital tract. **a** All tumors. **b** MLA of the endometrium. **c** MLA of the ovary

The overall frequencies of *KRAS*-mutations within MLA of the endometrium and ovary are presented in Fig. 3.

The details of *KRAS*-mutational events in relation to the specific tumor site (endometrium versus ovary) are summarized in Supplementary Table 2.

## Discussion

Mesonephric-like adenocarcinomas (MLA) are a recently described morphologic subtype of malignancies of the female genital tract sharing histopathologic, immunohistochemical and molecular characteristics of mesonephric carcinomas (Euscher et al. 2020; Mills et al. 2022; Kim et al. 2022; Howitt and Nucci 2018; McCluggage 2022). Unlike mesonephric carcinomas, they are not associated with mesonephric remnants (Euscher et al. 2020; Mills et al. 2022; Kim et al. 2022; Howitt and Nucci 2018; McCluggage 2022). Histopathologically, they display mixed morphologic features on H&E-staining (Fig. 1a–d (Mirkovic et al. 2018; Pors et al. 2018; Horn et al. 2020; Euscher et al. 2020)). On immunohistochemistry, MLA show negativity or limited positivity for estrogen receptor staining and positive staining for TTF-1, CD 10 (Fig. 1e–g) and GATA-3 in most cases as well as for calretinin in some cases (Mirkovic et al. 2018; Euscher et al. 2020; Park et al. 2022; McCluggage 2022).

Adenocarcinomas with mesonephric-like features mostly occur within the uterus (74.7%; 115/154), a minority are diagnosed within the ovary [25.3%; 39/154 (Deolet et al. 2021)]. A similar distribution was reported in a multi-intitutional study (Pors et al. 2021): 63.8% (44/69) were of endometrial and 36.2% (25/69) of ovarian origin. Single cases have been described in the fallopian tube (Xie et al. 2021) or mesocolon (Deolet et al. 2022) in association with endometriosis. There are no obvious histomorphologic/immunohistochemical differences between the tumors located in the uterus versus those within the ovaries in the cases reported until now (Mirkovic et al. 2018; McCluggage 2022; Deolet et al. 2021; Koh et al. 2022).

Ovarian MLA may be associated with endometriosis or other epithelial ovarian tumors (Seay et al. 2020; McCluggage et al. 2020; Chen et al. 2020; Chang et al. 2023).

Based on immunohistochemical and molecular findings (see below), it is suggested that MLA are Müllerian-derived pathogenetically and display morphologic mesonephric features, indicating a process of transdifferentiation (da Silva et al. 2021; Euscher et al. 2020; McCluggage 2022). The concept of transdifferentiation may be supported by the presence of non-malignant mesonephric-like proliferations seen in association with mucinous ovarian tumors and identical molecular alterations within the mucinous borderline tumor and associated ovarian MLA (Nilforoushan et al. 2023).

With respect to the molecular classification of endometrial carcinomas (Soslow et al. 2019), uterine MLA show p53 wild-type immunostaining, retained mismatch-repair protein expression (see Fig. 1h–j) and are not associated with a POLE-mutation (Kolin et al. 2019; Horn et al. 2020; Ma et al. 2022; Deolet et al. 2021) defining them as endometrial carcinomas with no special molecular profile [NSMP (Horn et al. 2020; Deolet et al. 2021; McCluggage 2022)]. Within ovarian MLA, they show a retained mismatch-repair protein expression (Koh et al. 2022) and p53 wild-type staining (Koh et al. 2022; Chen et al. 2020; Mirkovic et al. 2018).

Uterine MLA showed a significantly reduced progression-free survival in comparison to endometrioid endometrial carcinomas, even compared to FIGO high-grade (G3) ones [*p* < 0.001 (Kim et al. 2022)]. Patients with endometrial MLA represent a poor clinical outcome, and 60–80% of the patients will be affected by recurrent disease or will die of the tumor (Mirkovic et al. 2018; Euscher et al. 2020; Kim et al. 2022; Kolin et al. 2019; Pors et al. 2021). Compared to endometrioid and serous endometrial carcinomas, MLA showed the lowest median progression-free survival [183 versus 67.1 versus 18.2 months; *p* < 0.0001 (Euscher et al. 2020)]. Pulmonary involvement, detected in up to 60% of endometrial MLA, is the most common site of distant spread (Kim et al. 2021; Park et al. 2022; da Silva et al. 2021; Euscher et al. 2020). Hepatic metastatic spread is ten times higher in patients with endometrial MLA compared to endometrioid endometrial carcinomas and five times more frequent when compared to those with serous tumors (Pors et al. 2021; Euscher et al. 2020; Mao et al. 2020). Other uncommon metastatic sites are the brain, spleen, and vertebrae (Pors et al. 2021; Deolet et al. 2021). Although many cases of endometrial MLA may be associated with adverse prognostic factors, such as large tumor size, deep myometrial involvement, cervical stromal invasion, involvement of lymphatic and/or blood vessels, and retroperitoneal lymphatic spread (Na and Kim 2019; Park et al. 2022; Kim et al. 2022; Euscher et al. 2020), there is an increasing evidence that the presence of a mesonephric-like histology itself may represent an independent poor prognostic feature (Euscher et al. 2020; Pors et al. 2021; Al Nabhani et al. 2022).

For ovarian MLA, Koh et al. (2022) reported a disease-free survival of 24.5 months (*N* = 5), and Pors et al. (2021) described a progression-free survival of 68% and an overall survival of 71% in a multi-institutional study of 25 cases. Three patients with FIGO-stage IC2 who were treated with adjuvant chemotherapy (carboplatin plus paclitaxel) showed no evidence of disease (NED): one patient after 13 months (Chen et al. 2020), one after eight (Xie et al. 2021), and one additional patient after 13 months (Xie et al. 2021) of follow-up, respectively. Within the study of Deolet et al. (2022), including patients with different FIGO stages, 3/5 showed NED after a mean follow-up of 22.4 months (range 8–46 months), 1/5 recurred after 11 months, and one patient presented with a partial response in FIGO-stage IVB after polychemotherapeutic treatment at 8 month follow-up. That very limited data for ovarian MLA may suggest a more favorable prognosis when compared to uterine MLA, but further investigations are necessary addressing that feature.

Overall, the most frequent mutational event in MLA of the female genital tract is a *KRAS*-alteration (Mirkovic et al. 2018; Kolin et al. 2019; Na and Kim 2019; Horn et al. 2020; Ma et al. 2022; da Silva et al. 2021; Koh et al. 2022; Deolet et al. 2022).

25–30% of the *endometrial* MLA cases harbor additional mutational events within the *PTEN*-, *CTNNB1*-, and *ARID1A*-genes (Mirkovic et al. 2018; Kolin et al. 2019; Euscher et al. 2020; da Silva et al. 2021; Mills et al. 2022; Ma et al. 2022) which are also common mutational alterations in endometrioid endometrial carcinomas.

Within the group of non-MLA of the endometrium, about 17% represent *KRAS*-mutations and are associated with an improved prognosis compared to *KRAS*-wild-type carcinomas (Kolin et al. 2019). Because of the high frequency of *KRAS*-mutational events in MLA [see below (Mirkovic et al. 2018; Kolin et al. 2019; Ma et al. 2022; Koh et al. 2022)], it may be expected that endometrial MLA are associated with a favorable clinical outcome—but the contrary is the case (Euscher et al. 2020; McCluggage 2022).

About 10–25% of ovarian MLA habour a wide range of different additional mutational events beside the *KRAS*-alteration, including *CTNNB1, PTEN, NOTCH3, NRAS,* and *PIK3CA* (da Silva et al. 2021; Deolet et al. 2022).

There is a wide range of treatment approaches in patients affected by MLA of the female genitalia (Euscher et al. 2020; Chen et al. 2020; Deolet et al. 2021, 2022; Xie et al. 2021; Koh et al. 2022).

Within the review of Deolet et al. (2021), the majority of patients with *endometrial* MLA underwent a total hysterectomy with bilateral salpingo-oophorectomy and pelvic lymph-node dissection; in some cases, para-aortal lymph nodes were also removed (Mirkovic et al. 2018; Deolet et al. 2021; Horn et al. 2020). In ovarian MLA, the surgical procedure has rarely been reported. Within the informative cases, tumor debulking was performed including an omentectomy (Deolet et al. 2021; Chen et al. 2020; Koh et al. 2022). Patients with endometrial MLA and morphologic risk factors received adjuvant radiation. About one-fifth of the reprted endometrial as well as ovarian MLA were treated with carboplatin and paclitaxel postoperatively (Deolet et al. 2021; Chen et al. 2020). As mentioned above, both uterine and ovarian MLA are negative or show only limited positivity for steroid hormone receptors (see Fig. 1e (Mirkovic et al. 2018; Euscher et al. 2020; Horn et al. 2020; Chen et al. 2020; Kim et al. 2021, 2022; Koh et al. 2022; da Silva et al. 2021; Ma et al. 2022; Park et al. 2022; McCluggage 2022)), suggesting that hormonal treatment may not be effective in MLA. One case of endometrial cancer with a mixed endometrioid and MLA histology was treated with progesterone therapy and recurred 6 years later only with the MLA component (Yano et al. 2019). Another case, initially (mis-)diagnosed as a low-grade endometrioid type endometrial cancer received hormonal treatment (not further specified) and recured 17 months later in the liver (Euscher et al. 2020). Reviewing 60 cases, hormonal treatment was not reported for any MLA occurring in the ovary (Koh et al. 2022).

Until now, mesonephric-like histopathology has not been incorporated into clinical guidelines (Euscher et al. 2020; Chen et al. 2020; Kim et al. 2021; Deolet et al. 2021). Because of its aggressive clinical behavior, close oncologic follow-up is indicated (Euscher et al. 2020; Kim et al. 2022; Pors et al. 2021; Deolet et al. 2021), and chest imaging may be recommended because of the increased frequency of pulmonary spread after histopathological diagnosis of a mesonephric-like phenotype (Mills et al. 2022). Because of the aggressive behavior, Euscher et al. (2020) suggested that treatment algorithms used for high-grade endometrial carcinomas should be considered in cases with mesonephric-like histopathology, even if they present with low-stage disease.

Based on the published data, the optimal (neo-)adjuvant systemic treatment remains unknown. Using the morphomolecular approach for the diagnosis of MLA of the female genital tract [mixed morphology on H&E-staining and immunoexpression of mesonephric-like markers; see above, (Horn et al. 2020; Pors et al. 2021; McCluggage et al. 2022)], it has been shown that a *KRAS*-mutation is a common finding in MLA (Mirkovic et al. 2018; Kolin et al. 2019; Horn et al. 2020; Ma et al. 2022; da Silva et al. 2021; Koh et al. 2022; Deolet et al. 2022; McCluggage et al. 2020). For example, 92% of the uterine MLA (12/13) and 87% of the ovarian MLA (13/15) in the study of da Silva et al. (2021) harbored *KRAS* somatic mutations affecting the hotspot codons 12 and 13 of *KRAS* (G12D, G12V, G12C, G12A, and G13D).

However, a mutational analysis was either not performed or reported in all published cases of the female genital tract (e.g., Pors et al. 2021; Xie et al. 2021; Chang et al. 2023). Within the review of Deolet et al. (2021), mutational results were available for 32.5% of the ovarian and uterine MLA included in that study. Reviewing 60 ovarian MLA, *KRAS*-mutational status was reported for 46.7% (28/60) of the cases (Koh et al. 2022).

The frequency of *KRAS*-alterations and the distribution of the different mutational sites of the informative published cases of uterine and ovarian MLA are summarized in Fig. 3. After analyzing the first published cases, it was hypothesized that the p.G12yD-alteration may be more prevalent in ovarian MLA, while a p.G12V-alteration was more common in uterine tumors (Mirkovic et al. 2018). However, increasing evidence does not confirm any predilection of any mutational site in the different location of the MLA within the female genitalia (Fig. 3).

There are very limited data on a potential biological significance of the different sites of *KRAS*-alterations in gynecologic (mesonephric-like) tumors (Mirkovic et al. 2018; McCluggage 2022). In a subset of colorectal cancers, the p.G12C-variant may be associated with a more aggressive clinical behavior (Chida et al. 2021). In the present study, we summarized the prognostic data in correlation to different mutational sites within the *KRAS*-gene reported in the literature (Table S1, Fig. 2). Thirty cases of endometrial and only 11 cases of ovarian tumors included data on prognostic outcome in correlation to the mutational site. The lowest rates of recurrent disease and/or death of disease were reported for cases with a p.G12C-alteration and for tumors with morphologic characteristics of MLA and a *KRAS*-wild-type status in patients with endometrial tumors (Fig. 2). For the ovarian localization, 5 out of 11 patients recurred without any predilection of a *KRAS*-mutational site (Tab S1). There are currently not enough data to draw any valuable conclusions regarding the prognostic impact of the different mutational sites within the *KRAS*-gene.

In the case of Al Nabhani et al. (2022), the endometrial primary and the ocular disease depicted the same mutational event *KRAS*-p.G12D.

Two cases in the study of da Silva et al. (2021) showed an identical clonal *KRAS*-mutation in the endometrial primary and the abdominal recurrence (*KRAS*-p.G12V) and also within *KRAS*-p.G12D when a uterine MLA and its pulmonal spread were compared. An identical clonal *KRAS*-alteration was observed in one case of the "Leipzig cohort" in the endometrial primary and different metastatic sites (case number 5, see Table 1).

Pors et al. (2021) did not report details for mutational analyses comparing endometrial and/or ovarian primary and their recurrence or the metastatic disease.

The Kristen rat sarcoma (*KRAS*) gene is one of the most common mutational alterations in solid tumors (Yang et al. 2023) and occurs in approximately 22% (Forbes et al. 2011).

Oncogenic mutations of the *RAS*-gene are frequent in colorectal cancers, affecting about 40% of the cases, of which 85% refer to *KRAS* (Cherri et al. 2023), mostly at codon 12 (Li et al. 2018). In colorectal cancer patients, a *KRAS*-mutation occured at p.G12V in 26.4%, followed by p.G12D (19.2%), p.G13D [16.5% (Malapelle et al. 2021)], and p.G12C in 8.5% (Schirripa et al. 2020). In the present study, a p.G12V KRAS-alteration was most commonly seen within MLA of the female genital tract (36.5%; 44/121), followed by p.G12D (33%) and p.G12C (7.4%; for details, see Fig. 2).

Recently, targeted inhibitors of *KRAS* have offered a breakthrough for solid tumors (Yang et al. 2023; Skoulidis et al. 2021; Fakih et al. 2022; Strickler et al. 2023; Hong et al. 2020). Sotarasib is a specific and irreversible inhibitor of the GTPase-protein in p.G12C-*KRAS*-mutated cancers. Pre-treated colorectal cancer patients with progression of disease showed an objective response rate of 9.7% in a phase 2 trial (Fakih et al. 2022). Within a phase 3 trial of 345 patients with non-small cell lung cancer (NSCLC) who progressed after previous treatment with chemotherapy and/or checkpoint inhibition, an improved progression-free survival after treatment with sotarasib was seen compared to docetaxel (5.6 versus 4.5 months, hazard ratio 0.66; *p* = 0.0017 (de Langen et al. 2023)). In patients with chemotherapeutically pre-treated metastatic pancreatic cancer, sotarasib was associated with an objective response rate of 21% (Strickler et al. 2023). In a basket trial of metastatic solid tumors (pre-treated with a median of three lines of chemotherapy), there was a disease control rate of 88.1% in NSCLC and of 73.8% in colorectal cancer patients (Hong et al. 2020). Within that trial, one out of two patients with (non-mesonephric-like) endometrial carcinoma showed stable disease.

According to the present results, only a minority of MLA of the female genital tract may potentially be targetable by sotarasib because of p.G12C-alteration: 7.9% (7/89) with endometrial and 6.2% (2/32) with an ovarian site (for details, see Fig. 3b, c).

Until now, targeted therapy against a *KRAS*-mutation at p.12GC with sotarasib has only been approved for lung and colorectal cancer by the Federal Drug Administration (FDA) and European Medical Association (EMA) (Skoulidis et al. 2021; Fakih et al. 2022; de Langen et al. 2023). Adagrasib is another covalent selective inhibitor targeting p.G12C (Cherri et al. 2023). The bicyclic peptide KS-58 showed anti-cancer activity within the lung cancer cell line A427 and the pancreatic cancer cell line PANC-1, harboring a p.G12D-alteration (Sakamoto et al. 2020). Furthermore, KS-58 exhibited anti-cancer activity against PANC-1 xenografts in mice in that study.

Preclinical data targeting the molecule Son of Sevenless-1 (SOS-1), catalyzing the conformation of *KRAS,* may offer a possible pan-*KRAS* inhibition (Kessler et al. 2021) rather than a single mutation inhibition. Different approaches for *RAS*-targeting are reviewed by Erlanson and Webster (2021).

Abstracting the data of MLA of the female genital tract, mesonephric-like histology per se is associated with a poor prognostic impact. A *KRAS*-mutation is the most frequent molecular event and is seen in 92% of endometrial and 87% of ovarian tumors (da Silva et al. 2021). Within the *KRAS*-mutated cases, a p.G12C-alteration is seen in 7.9% of endometrial and 6.2% of ovarian MLA (see Fig. 3b, c and Supplementary Table 1). Therefore, targeted inhibition of *KRAS*-G12C may offer a potential treatment approach only in the minority of MLA of the female genital tract.

## Supplementary Information

Below is the link to the electronic supplementary material.

Supplementary file1 (DOCX 20 kb)

## Data Availability

Data are available on request from the corresponding author.
