# Supplementary material for: Mesonephric-like adenocarcinoma of the female genital tract: possible role of KRAS-targeted treatment—detailed molecular analysis of a case series and review of the literature for targetable somatic KRAS-mutations
Source: J Cancer Res Clin Oncol. 2023 Sep 5;149(17):15727–36. doi: 10.1007/s00432-023-05306-9 (PMC10620254; doi:10.1007/s00432-023-05306-9)
Supplement: Supplementary file 1 — Supplementary file1 (DOCX 20 kb) [file 432_2023_5306_MOESM1_ESM.docx]

Table S2: Molecular details of endometrial and ovarian mesonephric-like adenocarcinomas focusing on *KRAS*-alterations

**Localization of *KRAS*-mutation additional**

**Author number of cases p.G12A p.G12C p.G12D p.G12V others mutational events**

1. **Endometrium**

Mirkovic et al. (2018) 3/3^1^ 3 1/3 PIK3CA

Patel et al. (2018) 1/1 1

Na & Kim (2019) 10/11 2 2 6 9/10 ARID1A

Yano et al. (2019) 1/1 1

Kolin et al. (2019) 4/4^2^ 1 1 2 1/4 PIK3R1

McCluggage et al. (2020) 1/3 1

Dundr et al. (2020) 1/1 1 1/1 PIK3CA

Horn et al. (2020) 4/4^3^ 1 3

Euscher et al. (2020) 17/23 1 7 5 4/17 wt^4^ 1/17 PIK3CA

Deolet et al. (2021) 1/1 G13N 1/1 VUS^5^ of PIK3CA

Ma et al. (2022) 4/4 1 1 2/4 wt 3/4 VUS of PIK3CA

1/4 VUS of PTEN

Kim et al. (2021) 22/25 1 9 6 5/22 wt

Kim et al. (2022) 6/7 1 3 2 2/6 ARID1A

1/6 ATM

Mills et al. (2022) 2/2 1 1 1/2 ARID1A

Park et al. (2022) 8/12^6^ 1 1 2 1/8 G13C 4/8 TP53

3/8 wt

Al Nabhani et al. (2022) 1/1 1

Horn et al. (present new cases) 4/4 1 2 1 3/4 VUS of CTNNB1,

HFE, Jak2, TP53, EGFR, Her-2

---------------------------------------------------------------------------------------------------------------------------------------------------------------------------------------------------

1. **Ovary**

McCluggage et al. (2020) 1/4 1

Seay et al. (2020) 1/1 1/1 wt 1/1 VUS of ATM and

PALB-2

Kim et al. (2021) 1/1 1

Mirkovic et al. (2018) 4/4 4 2/4 PIK3CA

Chapel et al. (2018) 1/1 wt NRAS and BCOR

Dundr et al. (2020) 1/1 1 PIK3CA and CHEK2

daSilva et al. (2021) 15/15 5 8 2/15wt

Deolet et al. (2022) 4/4 2 1 1/4 wt 1/4 PIK3CA and PTEN

Koh et al. (2022) 4/5 1 1 4

^1^Number of informative cases regarding molecular alteration / number of cases within the individual study

^2^One case of endometrial mesonephric-like carcinosarcoma included

^3^Four cases of the present study were previously published focussing on clinicopathologic data

^4^wt = wild type

^5^VUS = variant of unknown significance

^6^In the study of Park et al. [2022] mutational results were not reported for the 35 uterine MLA included in that study. But details regarding KRAS mutational events were given for 12 cases of endometrial mesonephric-like carcinosarcomas. These data were included in the present table.

Table S1: Patient outcomes in correlation to the specific site of *KRAS*-mutational event in endometrial and ovarian mesonephric-like adenocarcinomas*

**Localization of *KRAS*-mutation**

**Author no of cases p.G12A p.G12C p.G12D p.G12V other sites**

1. **Endometrium**

Kolin et al. (2019) 4 PUL 149mo pelvic 29mo NED 18mo

DOD 100mo

Dundr et al. (2020) 1 NED 12mo

Deolet et al. (2021) 1 p.1G3N HEP 9mo

Ma et al. (2022) 4 DOD 24mo NED 56mo wt PUL 13mo

wt NED 8mo

Mills et al. (2022) 2 NED 36mo pelvic 24mo

Euscher et al. (2020) 17 HEP 18mo PUL 23mo PUL 23mo NOS pelvic 4mo

Pelvic 17mo PUL 26mo wt NED 74mo

PUL 84mo AWD 14mo wt NED 4mo

Pelvic 1mo NED 19mo wt PUL 13mo

Pelvic 1mo PUL 9mo

PUL 5mo

HEP 17mo

Horn present study 1 HEP 11mo

1. **Ovary**

Seay et al. (2020) 1 wt abdominal 18mo

Chapel et al. (2018) 1 wt NED 3mo

Dundr et al. (2020) 1 HEP 1mo

Koh et al (2022) 4 NED 53mo NED 11mo PUL & HEP 13mo

NED 21mo

Deolet et al. (2022) 4 NED 22mo abdominal 9mo

NED 28 mo

Pelvic 1mo

The time intervals are documented as time between surgery/ treatment to first event (recurrence, metastasis or death of disease)

^1^ The shortest time of the first event (regardless of metastatic disease or dead of the disease) was recorded for each individual case. For example, if pulmonary spread was recorded after 12 months, but the patient died after 22 months after initial diagnosis, the initial event (12 months period) was recorded for the present analysis.

AWD = alive with disease, DOD = dead of disease, HEP = hepatic metastasis, NED = no evidence of disease, NOS = pathogentic mutation of the *KRAS*-gene, but codon not available, pelvic = pelvic recurrency, PUL = pulmonary metastatsis, wt= wild type,
